# Prevalence and risk factors of kidney disease in urban Karachi: baseline findings from a community cohort study

**DOI:** 10.1186/1756-0500-7-179

**Published:** 2014-03-27

**Authors:** Ashar Alam, Farhana Amanullah, Naila Baig-Ansari, Ismat Lotia-Farrukh, Faisal S Khan

**Affiliations:** 1 Department of Nephrology, The Indus Hospital, Korangi Crossing, Karachi 75190, Pakistan; 2 Indus Hospital Research Center, The Indus Hospital, Korangi Crossing, Karachi 75190, Pakistan; 3 Interactive Research & Development, Suite 508, Ibrahim Trade Tower, Main Shahrah-e-Faisal, Karachi 75350, Pakistan

**Keywords:** Chronic kidney disease, Pakistan, Prevalence, South Asia

## Abstract

**Background:**

Chronic kidney disease (CKD) is being increasingly recognized as a leading public health problem. However, there are limited data available with respect to prevalence of CKD in Pakistan, a developing South Asian country. The study presents the baseline findings of prevalence and risk factors for adult kidney disease in a Pakistani community cohort.

**Methods:**

A total of 667 households were enrolled between March 2010 and August 2011 including 461 adults, aged 15 and older. Mild kidney disease was defined as estimated Glomerular Filtration Rate (eGFR) ≥60 ml/min *with* microalbuminuria ≥ 30 mg/dl and moderate kidney disease was defined as eGFR <60 ml/min (*with or without* microalbuminuria).

**Results:**

The overall prevalence of kidney disease was 16.6% with 8.6% participants having mild kidney disease and 8% having moderate kidney disease. Age was significantly associated with kidney disease (p < 0.0001). The frequency of diabetes, hypertension and smoking differed significantly among the three groups, i.e., no kidney disease, mild kidney disease and moderate kidney disease.

**Conclusion:**

Our study results suggest that the burden of kidney disease in this population is found considerable and comparable to neighboring developing countries. We believe that these results have critical implications on health and economics of these countries and due to the epidemic of diabetes, hypertension, cardiovascular disease, smoking and association with worsening poverty, further rapid growth is expected. There is an urgent need for early recognition and prevention strategies based on risk factors and disease trends determined through longitudinal research.

## Background

Non-communicable diseases (NCDs) present a significant global health challenge in the current century and have replaced communicable diseases as the most common causes of morbidity and premature mortality worldwide [1]. Initially, four NCDs (cardiovascular disease, cancers, chronic respiratory diseases and diabetes) were prioritized in the Global NCD Action Plan [2] endorsed by the World Health Assembly in 2008 but systematic reviews of various population based studies have now revealed the significance of chronic kidney disease as a separate entity requiring emphasis on prevention, early detection and treatment [3,4].

In developing countries including Pakistan, the burden of CKD is growing [5] and is exacerbated due to poor community awareness, a disproportionately higher burden of known CKD risk factors and poor access to renal replacement therapy [6]. In resource restricted settings, overcoming this scenario becomes further complex due to insufficient community-based data that could help with targeted prevention. Recent population based studies [7-9] from Bhopal, India found an incidence rate of 150 cases of end stage renal disease per one million population (p.m.p). There is no incidence data for kidney disease available from Pakistan. A recent survey from Karachi, Pakistan conducted on 300 adults ages 30 years and above showed some degree of reduced glomerular filtration rate (GFR) in 25.3% of the screened population, with 5% having a GFR <60 ml/min, [10] whereas an earlier survey showed that approximately 15% to 20% of screened adults 40 years of age or older had a reduced estimated GFR <60 ml/min [11,12].

The Indus Hospital Community Cohort (IHCC) was established in 2010 with the objective of establishing a unique ‘Framingham-like’ [13] Pakistani cohort to investigate the prevalence and risk factors for hypertension, obesity, diabetes, coronary artery disease, kidney disease and hepatitis B and C infection in a multi-ethnic, middle to low income population. The paper highlights the baseline findings of kidney disease in the adult cohort population, including prevalence and risk factors for kidney disease.

## Methods

The Indus Hospital is located in Karachi, Pakistan, a densely populated city with a population of over 20 million. The catchment areas population is approximately 2.5 million and for the baseline cross-sectional, two administrative areas from Indus Hospital's catchment population were chosen for enrolment of a random selection of cohort households. Six hundred and sixty-seven households were enrolled between March 2010 and August 2011 including 461 adults. Detailed methodology of the cohort have been published elsewhere [14].

### Study sample and design

The baseline survey included questionnaires, anthropometric measurements, physical examination as well as ultrasound and laboratory assessment. Apart from questions directly related to kidney disease, questions regarding family as well as personal history of diabetes, hypertension, coronary heart disease, hyperlipidemia, and stroke were also asked. Information on smoking, regular exercise, alcohol intake, dietary habits including extra-salt or fat intake were also asked. Ultrasound examination of the kidneys and urinary bladder were performed and the size of kidneys and presence of stones and cysts were noted.

Laboratory tests: Serum creatinine was measured using Kinetic Colorimetric Assay in alkaline medium on Hitachi 902 (Japan). The glomerular filtration rate (GFR) was estimated using the new 4-variable Modification of Diet in Renal Disease (MDRD) equation as follows:

$$\begin{array}{l} \text{GFR}=186\times {\left(\text{serum}\;\mathrm{Cr}\;\left[\text{mg}/\text{dl}\right]\right)}^{‒1.154}\times \text{ag}e^{‒0.203}\\ \;\times \left(0.742\;\text{if}\;\text{female}\right) \end{array}$$

Urine for microalbumin was measured using Solid Phase Sandwich Immunometric Assay on Nycocard Reader II (Norway). Mild kidney disease was defined as eGFR ≥60 ml/min with microalbuminuria ≥30 mg/dl and moderate kidney disease was defined as eGFR <60 ml/min (with or without microalbuminuria).

Blood sugar random (BSR) was measured using Enzymatic Colorimetric without deproteination on Randox RX Imola (Japan). The glycated hemoglobin (HbA1c) levels were measured by high performance liquid chromatography on D-10 Hemoglobin Testing System (France). As per American Diabetes Association 2010 recommendations, [15] any participant having HBA1C ≥ 6.5% or BSR ≥ 200 mg/dl or taking anti-hyperglycemic agent was labeled as a diabetic. Participants were tagged as having increased risk of diabetes if their HBA1C level was between 5.7% – 6.4% *without* anti-hyperglycemic agent.

Systolic and diastolic blood pressures were measured by using an automatic oscillometric method in the sitting position after at least five minutes rest. Measurement was performed thrice, and the mean of the readings was used in the analysis. Hypertension was classified on the basis of cut-off set by Joint National Commission Report 7 (JNC7) [16] and participants having systolic blood pressure level ≥ 140 mm Hg, diastolic blood pressure level of ≥ 90 mm Hg or taking anti-hypertensive agent were labeled as hypertensive.

Body Surface Area (BSA) was calculated according to the Dubois and Dubois formula:

$$\begin{array}{l} \mathrm{BSA}\;\left(m^{2}\right)=0.20247\times \text{height}\;{\left(m\right)}^{0.725}\\ \;\times \text{weight}\;{\left(\mathrm{kg}\right)}^{0.425}. \end{array}$$

Body mass index (BMI) was derived by dividing weight (in kg) by height squared (in m^2^). Abnormal BMI level was set as ≥ 25 kg/m^2^ both in males and females.

The serum levels of total cholesterol and high-density lipoprotein (HDL) cholesterol were determined enzymatically on Randox RX Imola (Japan). The participants were considered to have dyslipidemia if the total cholesterol level was >200 mg/dl, HDL cholesterol level <40 mg/dl or they were taking anti-hyperlipidemic agents.

### Statistical analysis

The data was entered and analyzed using SPSS version 17. Shapiro Wilk’s test was applied to check the normality of quantitative variables like age, blood sugar, cholesterol, BMI, etc. Mean ± SD or Median (IQR) was calculated for the aforementioned quantitative variables depending on the normality assumption. Analysis of variance (ANOVA) or Kruskil Wallis test was applied to compare the quantitative variables among various groups (no kidney disease, mild kidney disease and moderate kidney disease) as appropriate. Chi-square test was used to check association between categorical variables such as age, gender, echogenic, cyst, loss of CMD, stone etc. and various groups.

#### Multinomial regression

Initially participants were categorized into those having no kidney disease, mild kidney disease and moderate kidney disease. Both univariate and multivariable multinomial regression analyses were performed to assess the correlation of various factors that were significant in univariate analysis with the outcome variable (no kidney disease, mild kidney disease and moderate kidney disease).

#### Binary logistic regression

However, due to the samll number of study participants with mild and moderate kidney disease, we decided to dichotomize the participants into those with no kidney disease and those with kidney disease. Both univariate and multivariable binary logistic regression analyses were performed. Multivariable binary logistic regression analysis was performed to assess the correlation among both groups for various risk factors that turned out to be significant in univariate analysis.

### Ethical approval

This study was approved by the Institutional Review Board (IRB) of Interactive Research and Development (IRD). Written informed consent was obtained from all participants.

## Results

A total of 461 adults (15 years of age and older) took part in the IHCC baseline survey. However, due to refusal to give urine specimen, 111 of them (42 men and 69 women) did not have urinary microalbumin data and their kidney disease status could not be classified. The socio-economic statistics of those excluded were similar to those who were included in the analysis. The kidney disease status of the remaining 350 adults, 126 males (mean age: 35.2 ± 16.7 years) and 224 females (mean age: 34.0 ± 14.2 years) were grouped into three categories: no kidney disease, mild kidney disease and moderate kidney disease. Mild kidney disease was defined as eGFR ≥60 ml/min with microalbuminuria ≥ 30 mg/dl whereas moderate kidney disease was defined as eGFR <60 ml/min (with or without microalbuminuria).

### Prevalence of kidney disease and age & gender distribution

The overall prevalence of kidney disease was 16.6% (58/350 participants) with 8.6% participants with mild kidney disease and 8% with moderate kidney disease. Age, not gender, was significantly associated with kidney disease (p < 0.0001) (Table 1).

**Table 1:** Characteristics of study participants, by age, gender and kidney disease status

	**< 30 years; n (%)**	**30–50 years; n (%)**	**> 50 years; n (%)**	**Total cohort**	**p value**
**Gender**					0.04*
Male	71 (42.30)	59 (35.10)	38 (22.60)	168 (36)	
Female	133 (45.40)	120 (41.00)	40 (13.70)	293 (64)	
	**No kidney disease**	**Mild kidney disease**	**Moderate kidney disease**	**Total cohort**	**p value**
**Gender**					
Male	100 (79.40)	14 (11.10)	12 (9.50)	126 (36)	0.3
Female	192 (85.70)	16 (7.10)	16 (7.10)	224 (64)	
**Age**					
< 30 years	137 (46.90)	15 (50.00)	1 (10.60)	153 (43.70)	0.000**
30-50 years	124 (42.5)	6 (20.00)	12 (30.00)	142 (40.60)	
> 50 years	31 (10.60)	9 (30.00)	15 (53.60)	55 (15.70)	

*p value < 0.05, calculated by Chi-square test.

**p value < 0.0001, calculated by Chi-square test.

### Characteristics of the study participants among the groups

The baseline characteristics of the participants are shown in Table 2. The mean values observed for age, systolic and diastolic blood pressures, serum creatinine, blood sugar random, HbA1c and HDL cholesterol levels were significantly different among the three groups.

**Table 2:** Characteristics of study participants on basis of kidney disease status

	**No kidney disease**	**Mild kidney disease**	**Moderate kidney disease**	**Total cohort**	**p value**
Age (years); Median (IQR)	30 (22–40)^a^	30 (19.75-54.25)^b^	52.5 (46–64.75)^a,b,c^	31 (22–43)	0.000**
n	292	30	28	350	
BMI (kg/m^2^); Median (IQR)	21 (18.6-25.3)^a^	21.8 (18.5-27.4)^b^	23.9 (20.4-28.6)^c^	21.33 (18.64-25.5)	0.09
n	290	30	28	348	
SBP (mmHg); Median (IQR)	118.7 (110.3-128.0)^a^	123.7 (109.6-130.0)^b^	133.0 (122.3-158.0)^a,b,c^	119.67 (110.67-129.67)	0.000**
n	291	30	28	349	
DBP (mmHg); Median (IQR)	75.0 (69.0-82.3)^a^	77.5 (70.5-82.7)^b^	83.7 (73.9-96.4)^a,c^	75.7 (69.3-83.0)	0.004*
n	291	30	28	349	
Creatinine (mg/dl); Median (IQR)	0.9 (0.8-1)^a^	0.9 (0.8-1.1)^a,b^	1.3 (1.12-1.48)^a,c^	0.9 (0.8-1.1)	0.000**
n	292	30	28	350	
BSR (mg/dl); Median (IQR)	95 (87–106.25)^a^	98.5 (85.7-117.75)^a,b^	104.5 (91.5-141)^a,c^	96 (87–111)	0.03*
n	290	30	28	348	
HBA1C (%); Median (IQR)	5.3 (4.8-5.8)^a^	5.8 (4.9-6.02)^a,b^	5.65 (4.82-6.07)^a,c^	5.4 (4.8-5.8)	0.03*
n	288	30	28	346	
Total CL (mg/dl); Median (IQR)	155.5 (138–184)^a^	148.5 (128–177.25)^b^	164 (138.5-220.75)^c^	155 (137–184)	0.3
n	292	30	28	350	
HDL (mg/dl); Median (IQR)	40 (35–45)^a^	37.5 (31.75-42)^a,b^	42 (37–45)^a,c^	40 (34–45)	0.03*
n	292	30	27	349	

^a, b^^ & c^shows the significance difference between the groups. If any two or more groups have the same letters, there is significant difference between the groups.

*p value < 0.05 and **p value < 0.0001, calculated using Kruskil Wallis test.

IQR: Interquartile range (25th percentile – 75th percentile).

**Figure 1:**
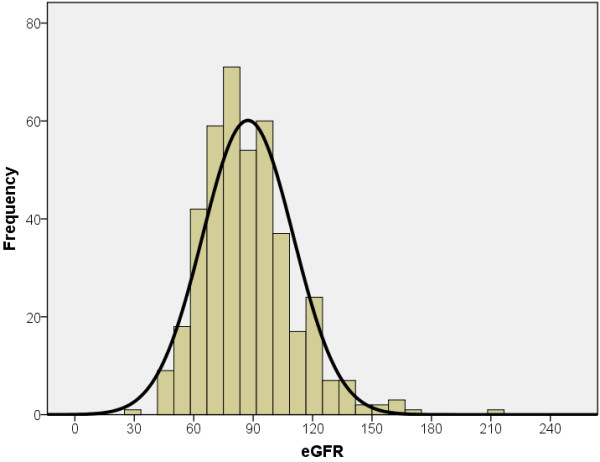
**Distribution of eGFR in study participants.** The histogram of eGFR from 415 study participants indicates a skewed distribution of the group with mean eGFR of 87.4 with a standard deviation of 22.9 mL/min/1.73 m^2^.

In the 415 participants where serum creatinine was available, the mean eGFR was 87.1 ml/min per 1.73 m^2^ (85.8 ± 23.5 ml/min per 1.73 m^2^ in males, and 87.8 ± 22.6 ml/min per 1.73 m^2^ in females). The eGFR distribution is shown in Figure 1.

### Relationship between abnormal variables, family history, lifestyle and kidney disease

The frequency with which diabetes and hypertension were noticed among the participants differed significantly among the three groups. However, dyslipidemia and abnormal BMI did not differ among the groups (Table 3). A statistically larger proportion of women (26%) were hypertensive as compared to men (16.4%) and a statistically smaller proportion of women (9%) were smokers as compared to men (18%) (Table 4).

**Table 3:** Frequency of abnormal variables on basis of kidney disease severity, n (%)

	**No kidney disease**	**Mild kidney disease**	**Moderate kidney disease**	**Total cohort**	**p value**
**Diabetes status**					
Diabetic	20 (6.80)	5 (16.70)	5 (17.90)	30 (8.60)	0.035*
Non-diabetic	272 (93.20)	25 (83.30)	23 (82.10)	320 (91.40)	
**Hypertension status**					
Hypertensive	53 (18.20)	7 (23.30)	13 (46.40)	73 (20.90)	0.002*
Normotensive	239 (81.80)	23 (76.70)	15 (53.60)	277 (79.10)	
**Dyslipidemia status**					
Dyslipidemic	157 (53.80)	18 (60.00)	15 (53.60)	190 (54.30)	0.8
Non-dyslipidemic	135 (46.20)	12 (40.00)	13 (46.40)	160 (45.70)	
**Body mass index (BMI), kg/m**^**2**^					
Underweight BMI < 18.5 kg/m^2^	79 (24.1)	7 (23.3)	5 (17.9)	82 (23.6)	0.9
Normal BMI 18.5-24.9 kg/m^2^	146 (50.3)	13 (43.3)	13 (46.4)	172 (49.4)	
Overweight (BMI 25.0-29.9 kg/m^2^	54 (18.6)	7 (23.3)	7 (25.0)	68 (19.5)	
Obese BMI ≥ 30 kg/m^2^	20 (6.9)	3 (10.0)	3 (10.7)	26 (7.5)	

*p value < 0.05; calculated by Chi-square test.

**Table 4:** Frequency of abnormal variables on the basis of gender

	**Male**	**Female**	**Total cohort**	**p value**
**Diabetes status**				
Diabetic	16 (10.4)	23 (8.5)	39 (9.2)	0.6
Non-diabetic	138 (89.6)	249 (91.5)	387 (90.8)	
**Hypertension status**				
Hypertensive	27 (16.4)	75 (26)	102 (22.5)	0.02*
Normotensive	138 (83.6)	214 (74)	352 (77.5)	
**Smoking status**				
Smokers	30 (18)	26 (9.0)	56 (12.3)	0.007*
Non-smokers	137 (82)	264 (91.0)	401 (87.7)	

*p value < 0.05; calculated by Chi-square test.

Smoking was significantly associated with kidney disease. Study participants either smoked regularly (12.3%) or were non-smokers (87.1%). Only three participants stated they were irregular smokers and for the purpose of analysis were considered non-smokers. We did not find a significant difference of water, salt and unsaturated fat intake between the groups (Table 5).

**Table 5:** Relationship between life style and kidney disease status

	**No kidney disease**	**Mild kidney disease**	**Moderate kidney disease**	**Total cohort**	**p value**
**Smoking status**					
Regular smoker	28 (9.60)	3 (100)	10 (35.70)	41 (11.70)	0.000**
Non-smoker	264 (90.40)	27 (90.00)	18 (64.30)	309 (88.30)	
**Water intake**					
Scanty, < 6 glasses a day	76 (26.00)	8 (23.30)	13 (46.40)	96 (27.40)	0.06
Adequate	216 (74.00)	23 (76.70)	15 (53.60)	254 (72.60)	
**Habit of adding salt**					
Yes	74 (25.30)	5 (16.70)	6 (21.40)	85 (24.30)	0.5
No	218 (74.70)	25 (83.30)	22 (78.60)	265 (75.70)	
**Unsaturated fat intake**					
Yes	76 (26.0)	4 (13.30)	6 (21.40)	86 (24.60)	0.3
No	216 (74.0)	26 (86.70)	22 (78.60)	264 (75.40)	

**p value < 0.0001, calculated by Chi-square test.

A family medical history of diabetes and hypertension was significantly associated with kidney disease (Table 6). However, a medical history of dyslipidemia, coronary artery disease, stroke, kidney stones, kidney failure, lower urinary tract symptoms, facial puffiness and pedal edema did not vary significantly among the three groups.

**Table 6:** Relationship between family medical history and kidney disease status of study participant

	**No kidney disease**	**Mild kidney disease**	**Moderate kidney disease**	**Total cohort**	**p value**
**Diabetes**					
Family history	58 (19.90)	7 (23.30)	0 (0.00)	65 (18.60)	0.03*
No family history	233 (80.10)	23 (76.70)	28 (100)	284 (81.40)	
**Hypertension**					
Family history	95 (32.60)	12 (40.00)	3 (10.70)	110 (31.50)	0.03*
No family history	196 (67.40)	18 (60.00)	25 (89.30)	239 (68.50)	
**Dyslipidemia**					
Family history	7 (2.40)	1 (3.30)	0 (0.00)	8 (2.30)	0.7
No family history	284 (97.60)	29 (96.70)	28 (100)	341 (97.70)	
**Coronary artery disease**					
Family history	39 (13.40)	3 (100)	0 (0.00)	42 (12.00)	0.1
No family history	252 (86.60)	27 (90.00)	28 (100)	307 (88.00)	
**Stroke**					
Family history	27 (9.30)	3 (100)	0 (0.00)	30 (8.60)	0.2
No family history	264 (90.70)	27 (90.00)	28 (100)	319 (91.40)	
**Kidney stones**					
Family history	39 (13.40)	3 (100)	2 (7.10)	44 (12.60)	0.6
No family history	252 (86.60)	27 (90.00)	26 (92.90)	305 (87.40)	
**Kidney failure**					
Family history	14 (4.80)	1 (3.30)	0 (0.00)	15 (4.30)	0.5
No family history	277 (95.20)	29 (96.70)	28 (100)	334 (95.70)	

*p value < 0.05; calculated by Chi-square test.

### Potential risk factors for kidney disease

We conducted univariate multinomial logistic regression analysis to determine parameters with a significant association with kidney disease (Table 7). Relationship with kidney disease was checked for age, HBA1C, gender, BSR, HDL, medical history of diabetes mellitus, medical history of hypertension as well as smoking status. None of the potential risk factors were statistically significant for mild kidney disease. However, factors found to be statistically significant in the moderate kidney disease group included increasing age (OR 1.1, 95% CI: 1.07-1.13), presence of diabetes (OR 3.0, 95% CI: 1.0-8.6), presence of hypertension (OR 3.9, 95% CI: 1.8-8.7), and being a regular smoker (OR 5.2, 95% CI: 2.2-12.4). The final multivariable multinomial logistic regression was built including only those factors that were statistically significant in univariate analysis. In the final multivariable multinomial model only age was statistically significant (OR: 1.08; 95 CI: 1.05–1.12).

**Table 7:** Univariate and multivariable risk factors for kidney disease status using multinomial regression

**Variables**	**Mild kidney disease**		**Moderate kidney disease**	
	**Prevalence odds ratio (95% CI)**	**p value**	**Prevalence odds ratio (95% CI)**	**p value**
***UNIVARIATE***				
**Age, yrs**	1.02 (0.99-1.04)	0.22	1.1 (1.07-1.13)	0.000**
**Diabetes mellitus status**				
Diabetic	2.7 (0.9-7.8)	0.07	3.0 (1.02-8.61)	0.047*
Non-diabetic	Ref		Ref	
**Hypertension status**				
Hypertensive	1.4 (0.6-3.4)	0.49	3.9 (1.8-8.7)	0.001*
Normotensive	Ref		Ref	
**Smoking status**				
Regular smoker	1.05 (0.3-3.7)	0.94	5.2 (2.2-12.4)	0.000**
Non-smoker	Ref		Ref	
**Gender**				
Male	1.7 (0.8-3.6)	0.18	1.4 (0.7-3.2)	0.4
Female	Ref		Ref	
**HDL**	1.011 (0.97-1.06)	0.62	0.95 (0.89-1.01)	0.09
***MULTIVARIABLE***	**Adj POR (95% CI)**	**p value**	**Adj POR (95% CI)**	**p value**
**Age, yrs**	1.01 (0.98-1.04)	0.49	1.08 (1.05-1.12)	0.000**
**Diabetes mellitus status**				
Diabetic	2.0 (0.4-9.6)	0.4	0.78 (0.16-3.9)	0.77
Non-diabetic	Ref		Ref	
**Hypertension status**				
Hypertensive	1.08 (0.4-2.9)	0.9	1.6 (0.6-4.1)	0.4
Normotensive	Ref		Ref	
**Smoking status**				
Regular smoker	0.9 (0.2-3.3)	0.9	2.6 (0.9-7.1)	0.07
Non-smoker	Ref		Ref	

The reference category is: absence of kidney disease.

*p value < 0.05; **p value < 0.0001 calculated by multinomial logistic regression.

*Abbreviation*: *POR* Prevalence Odds Ratio.

However, due to a paucity in the numbers of study participants with mild and moderate kidney disease, further analysis was done by dichotomizing kidney disease into two categories, i.e. those without kidney disease and those with kidney disease. Multivariate binary logistic regression was used to build models to explain the presence of kidney disease. Variables found significant at the univariate level or those with known biologically significance with kidney disease were used. Potential interaction between hypertension and smoking; hypertension and diabetes; as well as smoking and diabetes were explored. All interaction terms were found to be statistically significant and used accordingly in the models (Table 8). Three models were built with model 1 assessing the association between kidney disease, hypertension and smoking; model 2 assessing the association between kidney disease, hypertension and diabetes and model 3 assessing the association between kidney disease, smoking and diabetes (Table 9). Age was dichotomized into under 40 years and 40 years and above in keeping with biological significance.

**Table 8:** Univariate analyses of risk factors for kidney disease and interaction terms; binomial logistic regression

**Variables**	**Kidney disease**^**a**^	
	**Prevalence odds ratio (95% CI)**	**p value**
**Age** (for every 1 year increase in age)	1.05 (1.03-1.07)	0.000**
**Age groups**		
40+	4.0 (2.2-7.2)	0.000**
≤ 39	Ref	
**HDL**	0.96 (0.9-0.99)	0.03*
**Gender**		
Male	1.6 (0.9-2.8)	0.13
Female	Ref	
**Diabetes mellitus status**		
Diabetic	2.8 (1.3-6.4)	0.01*
Non-diabetic	Ref	
**Hypertension status**		
Hypertensive	2.3 (1.3-4.4)	0.006*
Normotensive	Ref	
**Smoking status**		
Regular smoker	2.7 (1.3-5.6)	0.007*
Non-smoker	Ref	
**Interaction terms**		
**Hypertension and smoking interaction**		
Normotensive & smoker	2.4 (0.98-5.8)	0.054
Hypertension & non-smoker	2.2 (1.097-4.4)	0.03*
Hypertension & smoker	8.9 (2.3-35.2)	0.002*
Normotensive & non-smoker	Ref	
**Hypertension and diabetes mellitus interaction**		
Normotensive & diabetic	2.0 (0.6-6.6)	0.2
Hypertension & non-diabetic	2.0 (1.006-4.1)	0.048*
Hypertension & diabetic	5.7 (1.8-18.0)	0.003*
Normotensive & non-diabetic	Ref|	
**Diabetes mellitus and smoking interaction**		
Non-diabetic & smoker	2.6 (1.2-5.8)	0.02*
Diabetic & non-smoker	2.7 (1.1-6.9)	0.04*
Diabetic & smoker	6.5 (1.3-33.4)	0.03*
Non-diabetic & non-smoker	Ref	

^a^. The reference category is: absence of kidney disease.

*p value < 0.05; **p value < 0.0001 calculated by binary logistic regression.

*Abbreviations* are: ***CI***-Confidence Interval, ***Ref***-reference.

**Table 9:** Multivariable analyses of risk factors for kidney disease using logistic regression; three explanatory models

**Models**	**Kidney disease**^**a**^	
	**aPOR (95% CI)**	**P value**
**Model 1:**		
**Age groups**		
40+ yrs	3.2 (1.7-6.0)	0.000**
<=39	**Ref**	
**Smoking and hypertension status**		
Normotensive AND smoker	1.6 ( 0.6-4.0)	0.35
Hypertensive AND non smoker	1.5 ( 0.7-3.2)	0.28
Hypertensive AND smoker	4.8 ( 1.1-20.3)	0.034**
Normotensive AND non smoker	**Ref**	
**Gender**		
Male	1.6 (0.8-3.0)	0.11
Female	**Ref**	
**Model 2:**		
**Diabetes and hypertension status**		
Normotensive AND diabetic	1.9 (0.58-6.2)	0.29
Hypertensive AND non diabetic	2.2 (1.1-4.5)	0.03*
Hypertensive AND diabetic	6.4 (2.0-20.5)	0.002*
Normotensive AND non diabetic	**Ref**	
**Gender**		
Male	1.8 (0.97-3.2)	0.06
Female	**Ref**	
**Model 3:**		
**Age groups**		
40+ yrs	3.4 (1.8-6.3)	0.000**
<=39	**Ref**	
**Diabetes and smoking status**		
Non diabetic AND smoker	1.8 (0.7-4.2)	0.2
Diabetic AND non smoker	1.6 (0.6-4.4)	0.3
Diabetic AND smoker	3.1 (0.6-16.4)	0.2
Non diabetic AND non smoker	**Ref**	
**Gender**		
Male	1.5 (0.8-2.8)	0.2
Female	**Ref**	

^a^. The reference category is: No Kidney Disease.

*p value < 0.05; **p value < 0.0001 calculated by logistic regression.

*Abbreviations* are: ***CI***-Confidence Interval, ***Ref***-reference.

Model 1- *Effect of hypertension and smoking on kidney disease*: The odds of older participants having kidney disease was a little over 3 times that of younger participants (aOR 3.2, 95% CI: 1.7-6.0) and of the odds of hypertensive smokers was almost five times that of participants who were normotensive and never smoked. (aOR 4.8 95% CI: 1.1-20.3); after adjusting for gender.

Model 2- *Effect of hypertension and diabetes on kidney disease*: The odds of kidney disease among hypertensive, non-diabetic participants was twice that of normotensive non-diabetics (aOR 2.2; 95% CI: 1.1-4.5); and over 6 times for hypertensive diabetic (aOR 6.4; 95% CI: 2.0-20.5), while adjusting for gender.

Model 3- *Effect of smoking and diabetes on kidney disease*: In the presence of diabetes, smoking status, age and gender, age was the only factor with a statistically significant association with kidney disease (aOR 3.4; 95% CI 1.8-6.3).

## Discussion

This is the first published study from Pakistan which has diagnosed kidney disease using albuminuria in addition to serum creatinine. Previous studies [10] have only used creatinine in their diagnosis.

Our study found the overall prevalence of kidney disease to be 16.6% with 8% participants having moderate kidney disease and 8.6% with mild kidney disease. In Karachi, a large metropolitan city of over 20 million people, two earlier community based surveys attempted to look at the prevalence of kidney disease. The first survey included 262 individuals >40 years of age with the same ethnic background and found a reduced GFR prevalence of 29.9% (defined as creatinine clearance <60 ml/min per 1.73 m^2^ measured in 24-hour urine collection) [11]. The other survey of 300 adults 30 years or older found that 25.3% had some degree of reduced eGFR based on serum creatinine, with 5% having eGFR <60 ml/min [10]. However, both these studies had not taken albuminuria into account which our study was able to do. An important study from Bangladesh involving 1000 participants from 15 to 65 years age, classified them on the basis of eGFR by MDRD equation and proteinuria on dipstick. The study found an overall CKD prevalence of 13.1% of which 6.6% had an eGFR ≥60 ml/min and proteinuria while 6.5% had an eGFR <60 ml/min [17]. This trend is similar to what we found in our study. Recently in Iran [18] and Thailand, [19] the overall prevalence of CKD was found to be 19.52% and 17.5% respectively with 10.63% and 8.9% having an eGFR ≥60 ml/min and an abnormal urine sediment while 8.89% and 8.6% with eGFR <60 ml/min. Similarly, the prevalence of CKD, (eGFR of <60 ml/ min), was found to be 8.4% in Japan [20] and 5.7% in Saudi Arabia [21]. Serial cross-sectional surveys over the last three decades demonstrate an increase in the prevalence of CKD [22-24]. Although the variation in CKD prevalence indicates differences between populations studied, an important contributor maybe the differences in calibration of serum creatinine assays and a lack of standardization across laboratories.

Our study found an overall estimated GFR of 87.1 ml/min per 1.73 m^2^ among all study participants, women were found to have a slightly higher eGFR. Moreover, only 40.6% of all participants had eGFR ≥90 ml/min per 1.73 m^2^ and only 10% had eGFR ≥120 ml/min per 1.73 m^2^. This is an interesting finding and necessitates studies aimed at validation of GFR in this particular population. Based on our findings, there is a possibility that “normal” values of GFR in our local population may be lower from those calculated in western countries.

In our study, gender was not found to be significantly associated with kidney disease. Earlier literature in this regard has shown variable results. Some studies [17,19-21] had not found a significant association between gender and CKD while others [3,24-26] found CKD to be significantly associated with the female gender. Interestingly enough, the risk factors of CKD such as coronary artery disease and smoking are more prevalent in males and are unlikely to explain the difference in CKD prevalence between genders. The gender disparity might partly be the result of an inaccurate correction factor for females in the GFR estimating equation or due to the differences in glomerular structure, glomerular homodynamics, diet, production and activity of local cytokines and hormones, and/or the direct effect of sex hormones, between genders [27]. Further investigation into the contribution of gender to CKD is required.

Age was found to be the most strongly associated risk factor in our study. Several studies performed in elderly populations have shown the prevalence of CKD to be more than 20% [28-31]. In general, GFR declines by 1 ml/min/1.73 m^2^ per year after the age of 30 years in healthy persons and the steep increase in the prevalence of CKD in the elderly might also be partly due to co-morbidities of CKD, such as cardiovascular diseases or diabetes, however, it is still unclear whether the decline in kidney function with increasing age represents pathology or is a part of the normal ageing process [32].

The present study showed that diabetes and hypertension were significantly more frequent in patients with kidney disease, however, dyslipidemia and abnormal BMI did not differ significantly among the study groups. Almost all the studies have shown similar trends with respect to diabetes and hypertension, but association of dyslipidemia and abnormal BMI with CKD is unclear. Studies from Japan [20,23] and Iran [26] have shown a strong association of these risk factors while other studies from Bangladesh [17], Iran [18], Thailand [19], and Saudi Arabia [21] have failed to show significant association in this regard. We feel as dyslipidemia and abnormal BMI are also associated with diabetes and hypertension, therefore, determining their association with CKD independent of these diseases may not be easy.

We could not find significant association between kidney disease and family histories of dyslipidemia, coronary artery disease, stroke, kidney stones, kidney failure, lower urinary tract symptoms, facial puffiness and pedal edema. A probable explanation of this might be the limitation of our study that all medical histories were self-reported. On the other hand, smoking, again self-reported was significantly associated with kidney disease in our study. The role of smoking as a risk factor for kidney disease is being increasingly recognized and similar findings have been noticed in our neighborhood Bangladesh [17]. Factors such as quantity of cigarettes being smoked need to be standardized to establish the association of smoking with CKD as an independent risk factor.

Finally, there was low awareness found in the general population with respect to non-communicable diseases like diabetes, hypertension and chronic kidney disease. This necessitates screening programs to be launched for early recognition and prevention of complications of these diseases, especially targeting CKD in high-risk populations. A clinical prediction score model can be developed to help in identifying high-risk populations.

### Study limitations

We were not able to use the term “chronic” kidney disease in describing out population since it is not possible to evaluate chronicity based on a single assessment. The definition of kidney disease used in the present paper was derived from the definition of Chronic Kidney Disease (CKD) first defined in 2002 Kidney Disease Outcome Quality Initiative (K/DOQI) Guidelines [33], and subsequently endorsed with minor modifications at the Kidney Disease: Improving Global Outcomes (KDIGO) Controversies Conference [34,35].

The glomerular filtration rate is ideally calculated through the measurement of urinary clearance of an ideal filtration marker such as insulin but is quite complex, expensive and difficult to perform in cohort studies. Therefore, we used an abbreviated version of the Modification of Diet in Renal Disease (MDRD) formula [36] to estimate GFR.

For evaluation of albuminuria, we measured microalbumin in a spot urinary sample and used a cut-off of ≥30 mg/dl to define albuminuria. In a recent update of CKD classification by KDIGO, [37] albuminuria has now been defined as an AER of ≥30 mg/24 hours (ACR≥30 mg/g [≥3 mg/mmol]) which is greater than three times the normal value in young adult men and women. Measurement of albumin to creatinine ratio (ACR) in a spot urinary sample was not possible in our study because of cost implications. Consequently, we categorized kidney disease into Moderate Kidney Disease with eGFR <60 ml/min irrespective of the level of proteinuria and Mild Kidney Disease with eGFR ≥60 ml/min and microalbuminuria ≥30 mg/dl in a spot urinary sample.

## Conclusion

Overall, this is the first published study from Pakistan that has diagnosed kidney disease using albuminuria in addition to serum creatinine. The burden of kidney disease in this population is found noteworthy and comparable to what have been seen in other developing countries of this region. We believe that these results have critical implications on health and economics of these countries. The rapid rise of common risk factors such as diabetes, hypertension and smoking especially among the poor, will result in even greater and more profound burdens that developing nations are not presently equipped to deal with. Moreover, there is a need to monitor risk factors and disease trends through longitudinal research. In conclusion, we feel there is a critical need for funding in developing countries to implement future community surveys followed by comprehensive, cost-effective and preventive public health intervention programs targeting chronic kidney disease.

## Competing interests

All the authors declared no competing interests.

## Authors’ contributions

AA and FA were responsible for the design of this study, the interpretation of the results and drafting the manuscript. NBA contributed to data analysis, critical review and statistical interpretation of the manuscript. ILK and FSK were responsible for the main cohort study field work and data acquisition. All authors read and approved the final manuscript.
